# Designing a Multi-Epitope Vaccine against *Toxoplasma gondii*: An Immunoinformatics Approach

**DOI:** 10.3390/vaccines10091389

**Published:** 2022-08-25

**Authors:** Mutiat Hammed-Akanmu, Maria Mim, Abdinasir Yusuf Osman, Abdulrahman M. Sheikh, Esmaeil Behmard, Ali A. Rabaan, Rapeah Suppain, Khalid Hajissa

**Affiliations:** 1 Department of Biomedicine, School of Health Sciences, Universiti Sains Malaysia, Kubang Kerian 16150, Kelantan, Malaysia; 2 The Royal Veterinary College, University of London, Hawkshead Lane, North Mymms, Hatfield AL9 7TA, UK; 3 Department of Medical Microbiology and Parasitology, School of Medical Sciences, Universiti Sains Malaysia, Kubang Kerian 16150, Kelantan, Malaysia; 4 School of Advanced Technologies in Medicine, Fasa University of Medical Sciences, Fasa, Iran; 5 Molecular Diagnostic Laboratory, Johns Hopkins Aramco Healthcare, Dhahran 31311, Saudi Arabia; 6 College of Medicine, Alfaisal University, Riyadh 11533, Saudi Arabia; 7 Department of Public Health and Nutrition, The University of Haripur, Haripur 22610, Pakistan; 8 Department of Zoology, Faculty of Science and Technology, Omdurman Islamic University, Omdurman P.O. Box 382, Sudan

**Keywords:** *Toxoplasma gondii*, epitopes, *in silico*, immunoinformatics, vaccine, molecular docking, immune simulation

## Abstract

Infection with the intracellular apicomplexan parasite *Toxoplasma gondii* causes serious clinical outcomes in both human and veterinary settings worldwide. Although approximately one-third of the world’s population is infected with *T. gondii*, an effective human vaccine for this disease remains unavailable. We aimed to design a potential *T. gondii* vaccine candidate that consisted of the B- and T-lymphocyte epitopes of three parasite immunogenic antigens. Firstly, the immunodominant epitopes expressed within the ROP2, MIC3, and GRA7 proteins of *T. gondii* were identified. Subsequently, six B-cell epitopes, five CTL epitopes, and five HTL epitopes were combined to generate a multi-epitope vaccine, and the 50S ribosomal protein L7/L12 was added as an adjuvant to boost the vaccine’s immunogenicity. All these epitopes were found to be antigenic, nonallergenic, nontoxic, and nonhuman homologs. The designed vaccine construct has a molecular weight of 51 kDa, an antigenicity score of 0.6182, and a solubility of 0.903461. Likewise, the candidate vaccine was immunogenic, nonallergenic, and stable. Molecular docking analysis revealed stable interactions between the vaccine construct and the TLR-4 immune receptor. Meanwhile, the stability of the developed vaccine was validated using molecular dynamics simulation. *In silico*, the vaccine construct was able to trigger primary immune responses. However, further laboratory-based assessments are needed to confirm its efficacy and safety.

## 1. Introduction

Toxoplasmosis is a life-threatening disease with medical, veterinary, and economic importance that is caused by the intracellular apicomplexan parasite *Toxoplasma gondii* [1,2]. The life cycle of *T. gondii* can be summarized broadly into two components: the sexual (which occurs only in felines, the definitive host: wild or domestic), and the asexual, which occurs virtually in all warm-blooded animals (the intermediate hosts) [3]. Infection pathways in humans include ingestion of tissue cysts, contamination with oocyst, and congenital infection [4]. Although individuals with competent immune systems are usually asymptomatic or present mild self-limiting symptoms, toxoplasmosis can result in serious and life-threatening complications in immunocompromised patients and congenitally infected children [5]. Furthermore, toxoplasmosis in animals is of great economic concern, particularly in small ruminant industry [6,7]. The disease causes abortion, stillbirth, and neonatal loss if it occurs in a pregnant animal [8]. Human populations are often at risk of toxoplasmosis, and vaccines against *T. gondii* remain a public health priority [9]. Thus far, Toxovax, the live attenuated *T. gondii* S4, is the only commercially available vaccine for animal use with the risk of associated adverse effects [10]. However, Toxovax cannot be employed in humans given its risk of reversion to virulence. 

To date, different vaccination approaches to *T. gondii* infection have been developed including the use of crude or recombinant parasite antigens, inactivated or live attenuated vaccines, subunit or multi-antigenic vaccines, and DNA vaccines [11]. However, the development of the vaccine using conventional approaches is not cost-effective [12]. Reverse vaccinology is the proposed new approach for developing novel vaccines that combine immunogenomics and bioinformatics. This approach has several advantages over traditional vaccination including efficiency in vaccine production time, safety, and cost-effectiveness associated with its production [13]. Bioinformatic approaches help investigate the whole spectrum of probable antigens using molecular modeling to investigate potential binding interactions with the host protein; the first step needed to develop a vaccine, which is the recognition of an immune protective antigen, can be achieved using bioinformatics [14,15]. In addition, immune-informatics has been shown to be helpful in predicting the antigenic peptide of B-cell and T-cell epitopes for the development of epitope-based vaccines [16]. Considering the complexity of *T. gondii*’s life cycle, immunization with *T. gondii* stage-specific antigens has been proven to provide stage-limited protection. Therefore, acquiring a multiepitope vaccine containing the B- and T-cell epitopes of different parasite life cycle stages is a promising strategy for preventing toxoplasmosis [17]. In this regard, immunization with a multiepitope vaccine against various pathogens has resulted in a notable increase in cellular and humoral immune responses along with prolonged survival time [18]. Thus, effective vaccines for protection against toxoplasmosis have been developed lately using bioinformatics that explores the potential of B- and T-cell epitopes. Accordingly, epitope-based vaccines have been shown to be potential candidates for developing novel and effective *T. gondii* vaccines [17,19,20].

In this study, robust immunoinformatics approaches were used to design a potential *T. gondii* multiepitope vaccine. Three *T. gondii* excretory–secretory antigens, namely, Micronemal protein 3 (MIC3), dense granule protein 7 (GRA7), and rhoptry protein 2 (ROP2), which play a major role in stimulating protective immunity were utilized. All these antigens are expressed in the three stages of *T. gondii* simulating protective cellular and humoral immunity against the parasite of interest [21,22].

## 2. Materials and Methods

### 2.1. Retrieval of the Parasite Protein Sequences

The full-length amino acid sequences of the selected *T. gondii* proteins, namely: MIC3 (accession no: A0A7J6KDBO), GRA7 (accession no: 17CQR1), and ROP2 (accession no: A0A7J6JZL4) were retrieved from the UniProt database at https://www.uniprot.org (15 February 2021) in FASTA format. Subsequently, the antigenicity of the three proteins was then predicted using the VaxiJen v2.0 server (http://www.ddg-pharmfac.net/vaxijen/VaxiJen/VaxiJen.html (20 February 2021)) with a default threshold value of 0.5.

### 2.2. Linear B Lymphocyte Epitope Prediction 

The linear B lymphocyte (LBL) epitopes of the target proteins were predicted using B-cell epitope prediction server (BCPREDS) available at http://ailab.ist.psu.edu/bcpred/predict.html (21 February 2021). This prediction server uses machine learning algorithms that differentiate between experimental and non-experimental B cell epitopes. The criteria such as 75% specificity and the use of overlap filters and the epitope length of 20 amino acids were used. The human homology of the selected epitopes was predicted using BLASTp server at https://blast.ncbi.nlm.nih.gov/ (25 February 2021). For this prediction, default parameters were used and Homo sapiens [taxid: 9606] were used for organism comparison. The e-value threshold was selected to be 0.05 and the epitopes less than the e-value were considered as being non-homologous peptides [23].

### 2.3. Prediction of Cytotoxic T-Lymphocytes Epitopes

For the identification of cytotoxic T-lymphocytes (CTL) epitopes, the retrieved amino acid sequences were analyzed using NetCTL 1.2 server (https://www.cbs.dtu.dk/services/NetCTL/ (10 March 2021)) at a threshold value of 0.75 [24]. Predicted epitopes were further assessed for their antigenicity via VaxiJen v2.0 [25] and MHC class I immunogenicity (http://tools.iedb.org/mhci/ (15 March 2021)) [26]. ToxinPred (http://crdd.osdd.net/raghava/toxinpred/ (17 March 2021)) was used to determine the toxicity [27], whereas AllergenFP V1.0 (http://ddg-pharmfac.net/AllergenFP/ (23 March 2021)) was used in the prediction of the allergenicity [28] and the IEDB conservation analysis resource (http://tools.iedb.org/conservancy/ (25 March 2021)) was applied to check the conservancy. The server’s default parameters were utilized for all of the above predictions.

### 2.4. Prediction of Helper T-Lymphocyte Epitopes 

The helper T-lymphocyte (HTL) epitopes expressed within the three target proteins were identified using the Immune Epitope Database MHC class II binding allele prediction tool available at http://tools.iedb.org/mhcii/ (30 March 2021). Three different MHC class II alleles including HLA-DQA1*05:01, HLA-DQB1*02:01, and HLA-DRB1*03:01 were employed as target alleles. CONSENSUS 2.22 method [29] was used and epitopes were chosen considering the 5% percentile rank. Allergenicity was predicted as mentioned elsewhere. Interferon-gamma was determined using IFNepitope. The predicted HTL epitopes were further assessed for antigenicity, toxicity, and allergenicity properties. In addition, the IFN epitope server (http://crdd.osdd.net/raghava/ifnepitope/ (10 April 2021)) was used to evaluate the ability of the HTL epitopes to stimulate IFN-γ production.

### 2.5. Designing of Multi-Epitope Vaccine Construct

Using specific peptide linkers, the final vaccine construct was designed using the LBL, CTL, and HTL epitopes that passed the selection criteria. The epitopes were linked together using AAY linker for CTL and KK linker for both HTL and LBL epitopes. The 50S ribosomal protein L7/L12 (NCBI ID: P9WHE3) was linked to the N-terminus of designed vaccine as an adjuvant by an EAAK linker. This helps in enhancing the amount of the antigen-specific immune response elicited. 

### 2.6. Physiochemical Properties, Allergenicity, Solubility, and Antigenicity Prediction of the Designed Vaccine 

The physicochemical parameters (number of amino acids, theoretical isoelectric point (pI) molecular weight, amino acid, and atomic composition, extinction coefficients, estimated half-life, aliphatic and instability index, as well as grand average of hydropathicity (GRAVY)) of designed vaccine construct were evaluated by submitting the primary protein sequence to ProtParam web-server (http://web.expasy.org/protparam/ (12 April 2021)) [30]. Protein instability index determines the protein’s stability and a stability index of 40 above indicates that the protein is unstable. VaxiJen 2.0 server was used in predicting the vaccine antigenicity. The method for predicting antigenicity was exclusively based on the proteins’ physiochemical properties with recourse to protein alignment with the precision rate between 70–89% [31]. Given, that an allergenic protein induces a harmful immune response, the AllergenFP servers were employed to predict allergenicity potential [32]. Solubility was predicted using SOLpro (http://scratch.proteomics.ics.uci.edu/ (12 April 2021)). 

### 2.7. Predicting Secondary Structure

The secondary structure of the designed vaccine construct was generated using PSIPRED online tool and SOPMA server. PSIPRED (http://bioinf.cs.ucl.ac.uk/psipred/) is one of the most widely used servers for predicting protein secondary structure. The server uses two feed-forward neural networks to analyze PSIBLAST-generated protein output with high accuracy. During the analysis, all server parameters were kept at their default values. The SOPMA server, a self-optimized prediction server, has a prediction accuracy of more than 80% [33]. 

### 2.8. Predicting the Tertiary Structure, Refinement, and Validation of the Designed Vaccine 

The final tertiary structure of the designed vaccine construct was modeled via RaptorX server available at http://raptorx.uchicago.edu/StructurePrediction/predict (20 April 2021) [34], the predicted model was refined via GalaxyRefined server, http://galaxy.seoklab.org/cgibin/submit.cgi?type=REFINE (20 April 2021) [35]. Next, the refined 3D model was validated using Ramachandran plot analysis, different quality factors, and error plot analysis via the PDBsum server (http://www.ebi.ac.uk/thorntonsrv/databases/pdbsum/Generate.html (20 April 2021)) [36]. The overall model quality that is displayed in context of Z-score, X-ray and NMR plot and local quality plot for the input structure were assessed via ProSA-web server available at https://prosa.services.came.sbg.ac.at/prosa.php. ERRAT server, http://servicesn.mbi.ucla.edu/ERRAT (20 April 2021) was used to analyze non-bonded atom–atom interaction [37]. 

### 2.9. Molecular Docking with Toll-Like Receptor (TLR) 

To determine the binding affinity of the designed vaccine construct with toll-like receptor (TLR-2, 6NIG, and TLR-4, 4G8A) and major histocompatibility complex (MHC class I, 2XPG, and MHC class II, 3C5J) present on the surface of human immune cells, molecular docking was conducted using the ClusPro 2.0 server (https://cluspro.bu.edu/login.php (5 May 2021)) [38]. The PDB-files of all the aforementioned receptors were obtained from the RCSB protein data bank and the multi-epitope vaccine was used as ligand. Efficiently docked complexes were chosen and downloaded based on the lowest energy score. The ClusPro server’s output was additionally modeled by PyMOL molecule graphic system version 2.0. 

### 2.10. Immune Simulation 

The probability of the designed vaccine inducing both humoral and cellular immune responses was further assessed using the C-ImmSim server (https://kraken.iac.rm.cnr.it/C-IMMSIM/ [39]. Three injections of the designed multi-epitope vaccine with time steps of 1, 84, and 168, corresponding to 0, 4, and 8 weeks, were used to simulate the immune response.

### 2.11. Molecular Dynamics Simulation

In order to assess the structural stability of the vaccine, construct-TLR-4 docked complex as well as the overall mobility analysis molecular dynamics simulation was conducted using GROMACS version 2020 [40]. Briefly, the OPLS-AA force field and TIP3P water model were used. The simulation system was then neutralized by genion module and the system’s energy was minimized using the steepest descent algorithm. The system’s temperature was gradually increased from 0 to 310 K during 0.5 ns, and the pressure was set at 1 bar.

Subsequently, in an NPT ensemble, 0.5 ns simulation was carried out at the pressure of 1 atm, and the temperature of 310 K. Production simulation for 100 ns was then implemented. The particle mesh Ewald (PME) and the LINCS algorithms were applied to assess all electrostatic connections and to restrain all bond lengths in the protein, respectively. Moreover, periodic boundary condition was utilized during the simulation. The final coordinates obtained for the complex system were analyzed with classic MD analyses.

### 2.12. Codon Optimization and In Silico Cloning

Codon optimization was carried out using Java Codon Adaptation Tool (JCat) server in order to maximize the production of the designed vaccine construct in an appropriate expression system [41]. The step is necessary because the degeneracy of the genetic code allows most amino acids to be encoded by multiple codons. The coding sequences of vaccine construct were codon-optimized for protein expression in *E. coli* (strain K12) host. The output of the (JCat) server includes two parameters: codon adaptation index (CAI) and the percentage of GC content. The ideal CAI score is 1.0; however, a score of 0.8 or above is also considered a good score, while the GC content should be between 30 and 70%. Finally, the codon-optimized sequence was then cloned into pET28a(+) expression vector through the addition of *EcoR*I and *BamH*I restriction sites at the N and C-terminals, respectively, using the SnapGene software. 

## 3. Results

### 3.1. Protein Retrieval and Antigenic Prediction

In this study, three *T. gondi* antigens, ROP2, GRA7, and MIC3, with lengths of 372, 249, and 383 amino acids, respectively, were obtained. Analysis of the antigenicity by the Vaxijen v2.0 server confirmed the antigenic nature of the three proteins is above the 0.5% threshold with corresponding values of 0.616, 0.5453, and 0.8553 for ROP2, GRA7, and MIC3, respectively. 

### 3.2. Linear B Lymphocyte Epitope Prediction

Preliminary prediction analysis of LBL epitopes using the BCPRED server revealed a total of 11, 6, and 12 epitopes from ROP2, GRA7, and MIC3, respectively. Further assessment revealed that only eight LBL epitopes met the pre-defined selection criteria, which included antigenicity, nontoxicity, non-allergenic, and non-human homologous (Table 1). Of these, only six epitopes we selected for the final vaccine design based on the antigenicity score. 

### 3.3. Cytotoxic T Lymphocyte Epitopes Prediction

Using the NetCTL v1.2 server, a total of 22 CTL epitopes with a length of 9 amino acids were predicted from the three target proteins. NetCTL v1.2 server uses default score 0.75. The epitopes selected in this study have a combined score of >0.75 [42]. Assessment of the immunogenicity of the epitopes reveals that only eight are immunogenic, of which five epitopes passed the other selection criteria (Table 2). All five CTL epitopes were included in the final vaccine construct in duplicate.

### 3.4. Helper T Lymphocyte Epitopes Prediction

A total of 478 HTL epitopes (15 mer in length) were identified using the IEDB server. The epitopes were selected based on the lowest percentile rank of ≤5% (having IC50 value < 50 nM), as epitopes with lower percentile ranks have higher binding affinity [38]. The epitopes were subjected to interferon-gamma (IFNγ) prediction, a cytokine with the ability to stimulate innate and acquired immune responses of the host such as macrophages and natural killer cells. IFN epitopes prediction server employs Motif and support vector machine (SVM) hybrid and IFNγ versus other cytokines as a prediction model. IFN server uses dataset which activates T helper cells via inducing and non-inducing MHC class II binder. After being assessed for their ability to induce cytokines, 5 HTL epitopes were considered for incorporation into the final vaccine construct (Table 3).

### 3.5. Designing Multiepitope Vaccine

**Figure 1:**
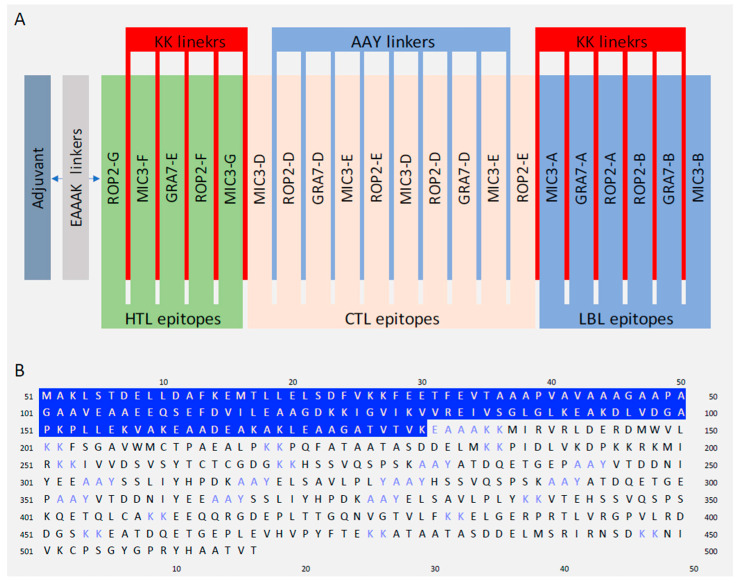
The final T. gondii multi-epitopes vaccine design. (**A**) A graphical presentation of the T. gondii multi-epitope vaccine design. The candidate vaccine construct containing (left to right) an adjuvant, HTL, CTL and LBL. (**B**) The primary structure of the designed vaccine in one letter format of amino acid, adjuvant (blue highlight), linkers (blue) and epitopes (black).

The final vaccine construct was designed to contain 21 immunogenic epitopes (5 HTL, 10 CTL and 6 LBL). These epitopes were linked together using appropriate linkers. Moreover, the 50S ribosomal protein L7/L12 as adjuvant was added to the construct’s N-terminal to improve the immunogenicity. The adjuvant was linked to the epitopes sequence by EAAAK linker. A schematic diagram of the final vaccine design is given in Figure 1.

### 3.6. Physiochemical Properties, Antigenicity, Allergenicity, and Solubility Prediction of the Designed Vaccine

The physicochemical properties of designed vaccine are presented in the Table 4. The molecular weight of the vaccine construct was predicted to be 50.35 kDa; with a theoretical pI 5.07; indicating its acidic nature. The instability index of the vaccine was estimated to be 37.51, indicating that it is a stable protein. In addition, the aliphatic index and GRAVY score were 73.87 and −0.485, respectively, showing hydrophobic vaccine nature. Moreover, the immunological potency of the vaccine was determined by assessing the antigenicity score. The vaccine was antigenic, with a score of 0.6182. In addition, the construct was soluble with a 0.829940 solubility score, and it is non-allergenic (Table 4).

### 3.7. Secondary Structure Prediction

The secondary structures: α helices, β-strands, and random coils were determined by two servers: PSIPRED and SOPMA. SOPMA predicted 41.58% α helices, 17.70% β-strands, and 40.72% random coils. The PSIPRED server, on the other hand, predicted 39.66 α helices, 11.73% β-strands as well as 51.39% random coils as shown in Table 5.

### 3.8. Tertiary Structure Prediction, Refinement, and Validation 

**Figure 2:**
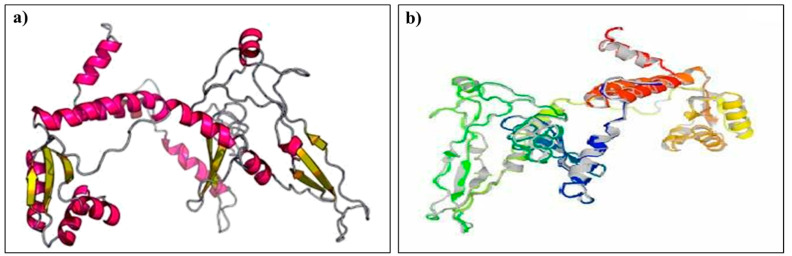
Prediction and refinement of the tertiary structure of the candidate vaccine. (**a**) The 3D model of the designed multi-epitope vaccine. (**b**) Galaxy-refined structure of the vaccine model.

**Figure 3:**
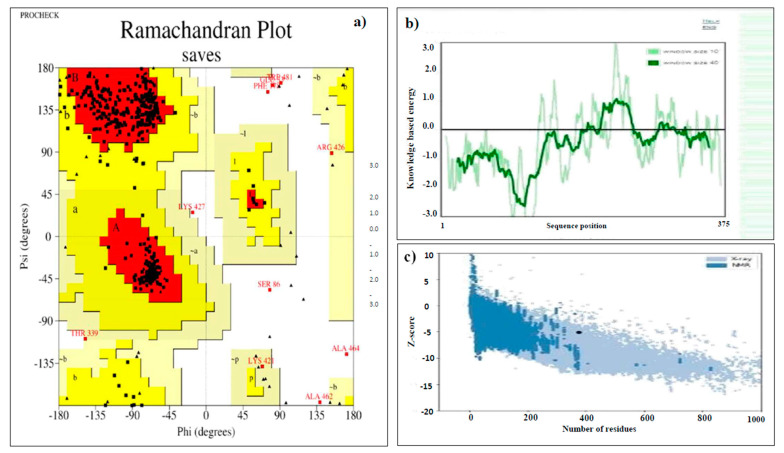
Validation of the 3D model of a multi-epitope vaccine. (**a**) Validation of the tertiary structure of the vaccine construct by Ramachandran plot, (**b**) local model quality of the vaccine construct. (**c**) Z-score of the vaccine construct as predicted by Pro server.

The 3D structure of the designed multi-epitope vaccine was modeled by the RaptorX server. The server generated five predicted models, of which model 4 was considered for further analysis (Figure 2a). The RaptorX predicted model was then refined using the Galaxy Refine server. Out of the five generated refined models, model 1 was chosen for additional analysis because it has the lowest RSMD score (0.296), GDT-HA score (0.9853), MolProbity score (2.062), a rotamers score of 0.4, and a Rama favored region of 94.1, indicating a better quality than the raw model (Figure 2b). The validation by Ramachandran plot analysis by the PROCHECKER server shows 90.7% residue in the most favored region, 6.8% residue in additional allowed regions, 1.0% residue in generously allowed regions, and 1.4% residue in disallowed regions as shown in Figure 3a. According to the ERRRAT server, the overall quality factor for the final model was 82.90. The model Z-score identified by ProSA was −5.16. The Q-mean value was calculated as −3.03 and the Q mean tool showed that the quality of the refined vaccine is likely within the range of the nonredundant set of PDB structures essential for protein with acceptable quality.

### 3.9. Molecular Docking with Toll-Like Receptor (TLR) 

**Figure 4:**
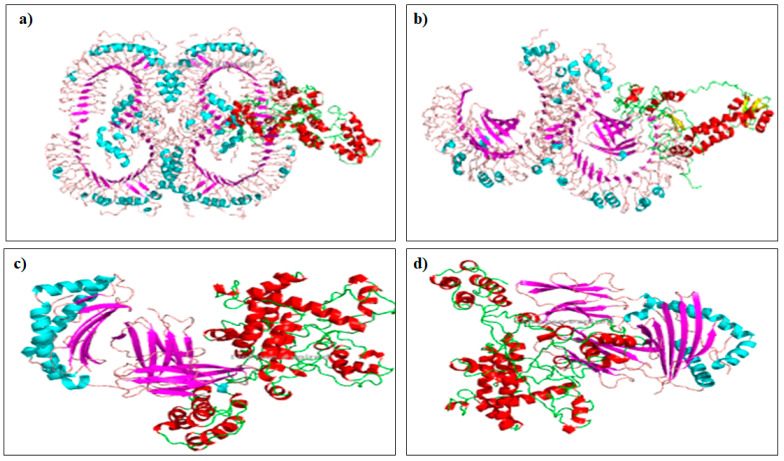
Protein–protein docking results of vaccine construct with TLR-2 (**a**), TLR-4 (**b**), MHC class I (**c**) and MHC class II (**d**).

Given the interaction between any vaccine candidate and the host immune receptor is essential for eliciting a protective immune response, molecular docking using ClusPro v2.0 server was conducted to determine the binding affinity of the multi-epitope vaccine with TLR-4 receptor. The server completes the task via three serial steps including rigid body docking, clustering of the lowest energy structure, and structural refinement by energy minimization. Human TLR-2 and TLR-4 had energy scores of 1095.1 and 886.3, respectively, and MHC class I and MHC class II had energy scores of −855.8 and −1049.0, respectively (Figure 4). Furthermore, Patchdock tools were employed for ranking the top ten interaction models between the immune cells and receptor model, and the best complexes were refined in Firedock. The vaccine and TLR4 complex were better bonds in solution ten, where the global energy was 3.87, van der Waals energy (vdW) was −45.54, repulsive energy was 68.59, atomic contact energy (ACE) was 1.81, and hydrogen bond energy was −4.00. 

### 3.10. Immune Simulation 

**Figure 5:**
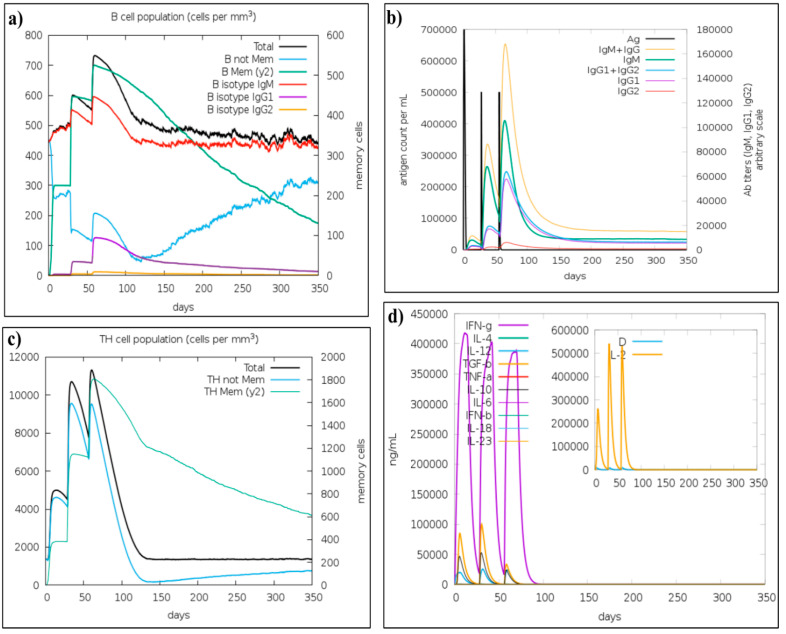
C-ImmSim presentation of an *in silico* immune simulation with the vaccine construct. (**a**) The generation of B-cells after the three injections. (**b**) Immunoglobulin production in response to antigen injections (various subtypes of immunoglobulin are represented as colored peaks). (**c**) The generation of Helper-T cells. (**d**) The cytokine profile.

The *in silico* simulations of immune responses resembled actual immunological responses triggered by infectious agents. For instance, the levels of immunoglobulin M (IgM) and immunoglobulin G (IgG) produced in the secondary and tertiary immune responses significantly higher in comparison to the primary response (Figure 5b). On the other hand, a strong cytokine response has also been observed. The IFN-gamma concentration was significantly higher (Figure 5d). Additionally, B-cell and T-helper populations were also increased with each injection (Figure 5a,c). Taken together, these findings confirmed the immunogenicity of designed vaccine construct. 

### 3.11. Molecular Dynamics Simulation

**Figure 6:**
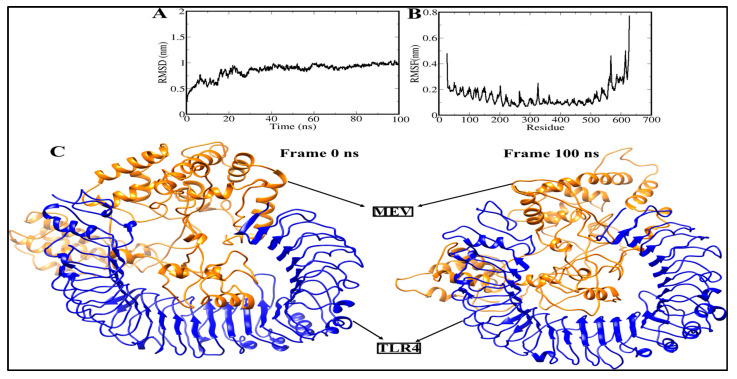
Structural stability analysis of simulated vaccine–receptor complexes: (**A**) RMSD and (**B**) RMSF. (**C**) The 3D view of multiepitope vaccine-TLR4 of frame 0 ns and frame 100 ns.

To evaluate the structural stability of the docked complex (vaccine construct-TLR4), some statistical features based on the 100 ns molecular dynamic simulation trajectory were analyzed and shown in Figure 6. The RMSD of Cα atoms in the vaccine construct-TLR4 complex gradually increases over time, this increasing trend continues until the time of 20 ns. The RMSD graph of Cα atoms then reaches a plateau, meaning that the vaccine construct-TLR4 complex mostly remained stable, and the RMSD value converged within ~0.1 nm. Additionally, the viewing conformations at varied time intervals clarified that the RMSF graph correlates to low structural changes created by the complex due to flexible loop regions. These changes had no effect on MEBV binding, nor on the total stability of the MEV-TLR4 complex. The MEV-TLR4 complex had a mean RMSD value of ~0.1 nm. The vaccine construct-TLR4 complex was next investigated by calculating the RMSF value of Cα atoms along a 100 ns MD simulation (Figure 6B). Periodic fluctuations in the Cα-RMSF values around 0.1–0.2 nm were seen in a 100 ns MD simulation. Our results confirmed that the vaccine construct has stable dynamics with the TLR4 receptor, meaning that the designed vaccine is strongly bound to TLR4.

### 3.12. Contribution of Energy Components to the Vaccine Binding along MD Simulations

**Figure 7:**
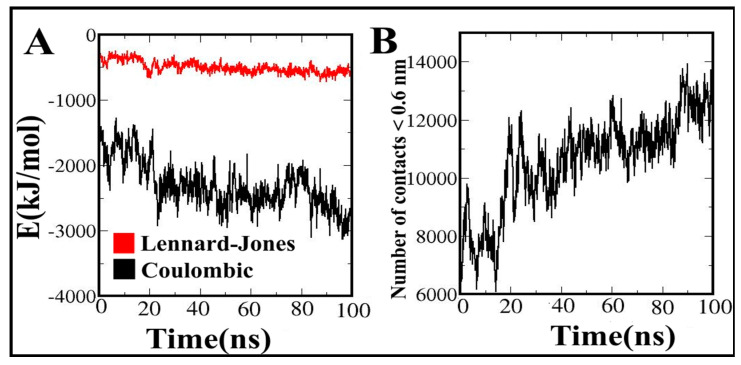
(**A**) Van der Waals, and electrostatic energies for the interactions of MEV with TLR4; (**B**) the number of contacts between the MEV and TLR4 during molecular dynamic simulation.

The interaction energies between the vaccine construct and TLR4 were estimated as separate electrostatic and van der Waals (vdW) components. The fluctuation template of these energies during the MD simulation were presented in Figure 7. Along MD simulation, the energies increased or fluctuated, and electrostatic energies reached up to −3000 kJ·mol^−1^, which shows that the vaccine constructs strongly bind to TLR4 (Figure 7A). The electrostatics component has a much greater share than vdW in the interaction with the vaccine during its MD simulations. Based on our results, electrostatic components play a major role in the vaccine construct binding as the main driving force. Accordingly, the number of contacts formed between the vaccine construct and TLR4 (Figure 7B) increases sharply at about 20 ns time point of the simulation, afterwards, the number of contacts between the vaccine construct and TLR4 slowly increases. These results indicated that the vaccine molecule strongly binds to the binding site of the TLR4. 

### 3.13. Codon Optimization and In Silico Cloning

**Figure 8:**
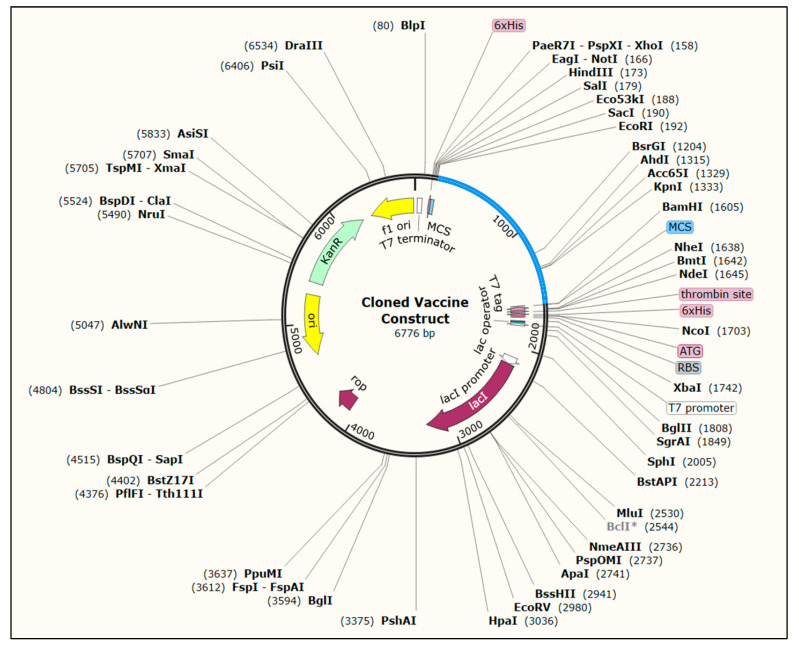
*In silico* cloning of the vaccine construct into pET28a(+) expression vector using SnapGene software. the red part represents the coding sequences of vaccine construct, and the black circle represents the vector backbone.

The codon-optimized cDNA sequence was estimated to be 1407 nucleotides in length. The optimized sequences had a CAI score of 0.99 and a GC content of 49.47%, indicating that the expression of the vaccine construct is efficient and potentially stable in the *E. coli* K-12 strain. The pET28a(+) expression vector containing the multi-epitope vaccine construct is represented in Figure 8. 

## 4. Discussion

Although acquiring a potential vaccine against *T. gondii* is needed to prevent the spread of infection in humans and animals, developing a protective toxoplasmosis vaccine remains a formidable challenge. The first step needed in developing a vaccine is recognizing immunoprotective antigens, which can be achieved using bioinformatics [43,44]. Bioinformatics helps to investigate the spectrum of potential antigens using molecular modeling to analyze the possible binding with the host protein. In addition, immunoinformatics aids in the prediction of antigenic peptide B-cell and T-cell epitopes for the development of peptide vaccines. Currently, immunoinformatics appears to be a promising strategy for successfully developing a broadly protective *T. gondii* vaccine. Multi-epitope vaccines have attracted increased attention in the field of vaccine development given their various advantages, which include high specificity, good safety, stability, and easy production and storage. It has been used to develop vaccines against a variety of infectious diseases, including toxoplasmosis [45,46,47], schistosomiasis [48], tuberculosis [49], mucormycosis [50], and coronavirus [51]. In this study, a robust immunoinformatics approach was used to design a novel multi-epitope vaccine against *T. gondii*. Firstly, the amino acid sequences of the target proteins (ROP2, GRA7, and MIC3) were retrieved from the UniProt database. The retrieved sequences were then submitted to the VaxiJen server to determine their antigenicity scores using the default server threshold values. The allergenicity scores of these proteins were above the threshold, thus making them eligible for inducing a strong immune response in the human body. The potential T-lymphocyte and linear B-lymphocyte epitopes were predicted and HTL (CD4+) is involved in activating the plasma B-lymphocytes that produce antibody and memory B-lymphocytes. It also activates the macrophages and CTL (CD8+) responsible for destroying the target antigen or infectious agents [31]. Researchers have reported that epitopes have to be accessible to MHC I and MHC II to produce the desired immune response [52]. The B-lymphocyte epitopes are important for inducing humoral and antibody-mediated immunity, as well as for activating the cells of the immune system. Six LBL, five CTL, and five HTL epitopes that passed the allergenicity, antigenicity, and toxicity tests were included in the final vaccine design. The epitopes were ensured to be nonhuman homologs considering that human homologs might induce autoimmune diseases. The multiepitope vaccine was constructed by connecting the five HTL, ten CTL, and six LBL epitopes successively using the KK, AAY, and KK linkers, respectively. Linkers are used in vaccine construction as indispensable elements because they aid the folding, expression, and stability of the vaccine [53]. The CTL epitopes were conjugated using the AAY linker to influence protein stability by providing the proteasomal cleavage site; enhancing epitope presentation and reducing immunogenicity [36]. Furthermore, KK (bi-lysine) linkers were used to conjugate the LBC and HTC epitopes to help preserve the epitopes’ independent immunogenic activities [54]. An adjuvant was used to construct the multi-epitope vaccine because the application of an adjuvant enhances antigen immune response, vaccine stability, and longevity compared with the use of T- and B-cell epitopes without an adjuvant [55]. The 50S ribosomal protein L7/L12 was utilized as an adjuvant because it enhances the immunogenicity of constructed vaccines [27]. The adjuvant was connected with the protein sequence using the EAAAK linker, which is rigid and minimally interferes with the vaccine antigenicity [56]. Finally, a vaccine with a length of 469 amino acids was constructed, as a vaccine with this length can be easily synthesized and mass-produced. 

The antigenicity and allergenicity results revealed that the vaccine construct was antigenic and nonallergenic and thus has the potential to be used. The vaccine construct had a molecular weight of 51 kDa, which is in line with the optimum molecular weight of the vaccine (>~40–50 kDa). Indeed, small molecules are typically removed from tissues by the blood, whereas proteins have increasing lymphatic absorption efficiency with increasing molecular weight. Thus, the likelihood of coming into contact with T and B lymphocytes in the lymph nodes increases [57]. The vaccine construct had a theoretical PI of 5.46, which indicated that the vaccine was acidic due to the presence of the acidic amino acids Asp and Glu. This characteristic is beneficial for protein purification through ion exchange chromatography and polyacrylamide gel electrophoresis. As a result, the vaccine construct’s solubility was anticipated using a solubility evaluating tool to determine the construct’s quality of being solvable inside the *E. coli* host, and the vaccine construct was confirmed to be solvable. Another key feature of a vaccine is its solubility, which was predicted to assess the vaccine construct’s solvability within the *E. coli* host, and the designed vaccine was found to be solvable (0.903461) inside the *E. coli* expression host. An aliphatic index of 77 (>70) suggests that a vaccine is thermostable over a wide temperature range [43]. A negative grand average hydropathy value indicates that the vaccine is hydrophilic, which has a good contribution to a water-based milieu [43]. The hydrogen bond structure between the amino hydrogen and carboxyl oxygen atoms in polypeptide chains represents the secondary structure, which typically consists of α-helices and β-structures [43]. The conformation of the protein structure is preserved by the presence of α-helices and β-turns inside the protein structure; these structures have high hydrogen bond energy that enables good interactions with antibodies [43,44]. 

The analysis of the secondary structure of the vaccine using the SOPMA server predicted that the vaccine comprised 41.58% α-helices, 17.70 % β-strands, and 40.72% random coils. In contrast, the PSIPRED server predicted that the vaccine contained 39.66% α-helices, 11.73% β-strands, and 51.39% random coils. The high percentage of α-helices and β-strands in the vaccine ensured high-energy hydrogen bonding, which helped maintain the structure of the protein and thereby ensured strong antibody interaction. However, a linear sequence is insufficient for predicting the immunogenicity and complexes of MHC and T-cell receptors, which are dependent on the spatial structure of the protein, and thus the 3D structure was predicted using RaptorX. The model with the highest RSMD score, which is indicative of superior 3D quality, was chosen for further refinement on the Galaxy server. Structure prediction helps determine the structure that is closely related to the vaccine protein structure, and 3D structure refinement helps reach parity with experimental accuracy [8]. Galaxy refinement helps rebuild and repackage side chains in the 3D model, thus enhancing the overall quality of the molecular dynamic simulation [29].

The refined model 1 was chosen in consideration of several quality parameters, such as GDT-HA (0.9853), RMSD (0.296), MolProbity (2.062), clash score (14.4), poor rotamers (0.4), and Rama favored (94.1). The global quality of the protein model was determined using GDT-HA, and RMSD was used to determine the deviation in the angles and bond lengths in the crude and refined models. Therefore, the best-refined model had a lower RSMD than the initial structure. Furthermore, a model with a lower MolProbity score than crystallographic resolution has better quality than the average structure at the same resolution [45,46]. The validation of the 3D structure through Ramachandran plot analysis on the PROCHECK SERVER showed that 90.8% of the amino acid residues were in the most favored region. The structure was predicted by the ERRAT tool to have an overall quality score of more than 82.9%; a score of more than 16 meets the generally acceptable score of >50% for high-quality models [47]. The Q-mean value was calculated to be −3.03, and the Q mean tool showed that the quality of the refined vaccine was likely within the range of a nonredundant set of PDB structures that are essential for obtaining a protein with acceptable quality. Moreover, VERIFY 3D showed that 58.676% of the amino acids had an average 3D–1D score ≥ 0.2. 

Molecular docking is a computational method that is used to determine the interactions between ligand and receptor molecules that are necessary to provide a stable adduct. In this study, TLR4 was used to dock our vaccine construct because it is one of the TLRs that are commonly found on the surfaces of cells. Several online servers, such as Cluspro 2.0, PatchDock, and FireDock, were used to increase the precision of our prediction. TLRs are members of the family of conserved pattern recognition receptors, which recognize specific pathogen patterns and can differentiate between self and nonself materials. The activation of TLRs by specific ligands induces cytokine production and MHC molecule upregulation. Finally, the innate immune response is linked to the adaptive immune response. The deformability region and high eigenvalue of the vaccine construct indicated that the chance of deformation amongst the molecules of the vaccine is low [40].

Strengths of this study include the use of robust immunoinformatics approaches as well as up-to-date immunological information, both of which are essential for vaccine design. The vaccine was designed based on LBL, CTL, and HTL epitopes with an N-terminal adjuvant, and immunoinformatics analysis revealed that our vaccine construct had satisfactory scores on the parameters of interest including antigenicity, allergenicity, toxicity, and physicochemical properties. The immune simulation measures showed their potential to trigger and induce a broad and potent immune response. Despite the fact that the current study is based on an integrated computational pipeline, the lack of in vivo and in vitro evaluation of the designed vaccine is a key limitation of this study. Therefore, the protective efficacy of the vaccine constructs against *T. gondii* infection as well as its safety should be validated through further experimental assessment. 

## 5. Conclusions

A potential multiepitope vaccine against *T. gondii* was designed using various immunoinformatics tools. The vaccine construct had satisfactory antigenicity evolution, physiochemical properties, allergenicity, toxicity, solubility, and immunogenicity. Molecular docking and molecular dynamics simulation analysis revealed that the vaccine construct–TLR-4 receptor interactions were stable. Future study is required to determine the protective efficacy of the vaccine construct against *T. gondii* infection as well as its safety. Further assessment in which the choice of antigens is based on biological criteria is urgently required.

## Figures and Tables

**Table 1:** LBL epitopes selected for vaccine construction.

Protein	Position	Name	LBC Epitopes	Antigenicity	Allergenicity	Toxicity	Human Homology
**ROP2**	56–75	ROP2_A	ELGERPRTLVRGPVLRDDGS	0.6399	Non-allergen	Non-toxic	Non-human homology
	80–99	ROP2_B	EATDQETGEPLEVHVPYFTE	0.7908	Non-allergen	Non-toxic	Non-human homology
**GRA7**	166–185	GRA7_A	EEQQRGDEPLTTGQNVGTVL	0.9203	Non-allergen	Non-toxic	Non-human homology
	24–43	GRA_B	ATAATASDDELMSRIRNSDF	1.0281	Non-allergen	Non-toxic	Non-human homology
**MIC3**	58–77	MIC_A	VTETHSSVQSPSKQETQLCA	0.8003	Non-allergen	Non-toxic	Non-human homology
	334–353	MIC_B	NIVFKCPSGYHPRYHAHTVT	0.6133	Non-allergen	Non-toxic	Non-human homology

**Table 2:** The CTL epitopes used in final vaccine construct.

Protein	Position	Name	CTL Epitopes	C-Score	Immunogenicity	Antigenicity	Allergenicity	Toxicity	Conservancy	Human Homology
ROP2	269–277	ROP2_D	ATDQETGEP	0.7645	+	0.6994	Non-allergen	Non-toxic	Conserved	Non-human homology
	545–553	ROP2_E	ELSAVLPLY	1.1063	+	0.5766	Non-allergen	Non-toxic	Conserved	Non-human homology
GRA7	111–119	GRA7_D	VTDDNIYEE	0.7539	+	0.6984	Non-allergen	Non-toxic	Conserved	Non-human homology
MIC3	62–70	MIC3_D	HSSVQSPSK	1.2568	+	1.355	Non-allergen	Non-toxic	Conserved	Non-human homology
	131–139	MIC3_E	SSLIYHPDK	0.8854	+	0.6068	Non-allergen	Non-toxic	Conserved	Non-human homology

**Table 3:** HLT epitopes selected for vaccine construction.

Protein	Position	Name	HTL Epitope	IFN γ	IL-4	IL-10	Antigenicity	Allergenicity	Toxicity	Conservancy	Human Homology
ROP2	81–95	ROP2_E	GSWLEQEAAEEVTPL	+	-	-	0.8870	Non-allergen	Non-toxic	conserved	Non-human homology
	325–339	ROP2_F	PIDLVKDPKKRKMIR	+	+	+	1.0777	Non-allergen	Non-toxic	conserved	Non-human homology
GRA7	336–360	ROP2_G	KMIRVRLDERDMWVL	+	+	+	1.0062	Non-allergen	Non-toxic	conserved	Non-human homology
	21–35	GRA7_E	PQFATAATASDDELM	+	-	-	0.6795	Non-allergen	Non-toxic	conserved	Non-human homology
MIC3	14–28	MIC3_E	FSGAVWMCTPAEALP	+	-	-	0.5938	Non-Allergen	Non-toxic	conserved	Non-human homology
	205–219	MIC3_F	IVVDSVSYTCTCGDG	+	-	-	1.1324	Non-allergen	Non-toxic	conserved	Non-human homology

**Table 4:** Antigenic, allergenic, and physiochemical characteristics of the construct.

Characteristics	Finding	Remarks
Number of amino acids	469	Suitable
Molecular weight	51,035.79	high
Theoretical PI	5.46	Acidic
Chemical formula	C2257H3602N598O719S13	-
Extinction coefficient (at 280 nm in H20)	39,560	-
Estimated half-life (mammalian reticulocytes, in vitro)	30 h	-
Estimated half-life (yeast, in vivo)	>20 h	-
Estimated half-life (*E. coli*, in vivo)	>10 h	-
Instability index of vaccine	37.53	Stable
Aliphatic index of vaccine	77.08	Thermostable
Grand average of hydropathicity (GRAVY)	−0.439	Hydrophilic
Antigenicity	0.6182	Antigenic
Allergenicity	No	Non-Allergen
Solubility	0.903461	Soluble

**Table 5:** The secondary structure of the vaccine construct.

	SOPMA Server		PSIPRED Server	
Features	Amino Acids	Percentage	Amino Acids	Percentage
Alpha helix	195	41.58	186	39.66
Beta strand	83	17.70	55	11.73
Random coil	191	40.72	241	51.39

## Data Availability

All data generated or analyzed during this study are included in this article. Other data will be available from the corresponding author upon request.
